# Prevalence, antimicrobial resistance profile, and characterization of multi-drug resistant bacteria from various infected wounds in North Egypt

**DOI:** 10.1016/j.sjbs.2022.01.015

**Published:** 2022-01-15

**Authors:** Mohamed A. Hassan, Sarah Abd El-Aziz, Horeya M. Elbadry, Samy A. El-Aassar, Tamer M. Tamer

**Affiliations:** a Protein Research Department, Genetic Engineering and Biotechnology Research Institute (GEBRI), City of Scientific Research and Technological Applications (SRTA-City), New Borg El-Arab City, P.O. Box: 21934, Alexandria, Egypt; b Polymer Materials Research Department, Advanced Technology and New Materials Research Institute (ATNMRI), City of Scientific Research and Technological Applications (SRTA-City), New Borg El-Arab City, P.O. Box: 21934, Alexandria, Egypt; c Botany and Microbiology Department, Faculty of Science, Alexandria University, Alexandria, Egypt

**Keywords:** Multidrug-resistant bacteria, Wound infections, Methicillin-resistant coagulase-negative *staphylococcus haemolyticus* (MRCoNS), *Klebsiella pneumoniae* and *Pseudomonas aeruginosa*, Extended-spectrum *β*-lactamases (ESBLs) bacteria

## Abstract

Multi-drug resistant (MDR) bacteria associated with wounds are extremely escalating. This study aims to survey different wounds in Alexandria hospitals, North Egypt, to explore the prevalence and characteristics of MDR bacteria for future utilization in antibacterial wound dressing designs. Among various bacterial isolates, we determined 22 MDR bacteria could resist different classes of antibiotics. The collected samples exhibited the prevalence of mono-bacterial infections (60%), while 40% included poly-bacterial species due to previous antibiotic administration. Moreover, Gram-negative bacteria showed dominance with a ratio of 63.6%, while Gram-positive bacteria reported 36.4%. Subsequently, the five most virulent bacteria were identified following the molecular approach by 16S rRNA and physiological properties using the VITEK 2 automated system. They were deposited in GenBank as *Staphylococcus haemolyticus* MST1 (KY550377), *Pseudomonas aeruginosa* MST2 (KY550378), *Klebsiella pneumoniae* MST3 (KY550379), *Escherichia coli* MST4 (KY550380), and *Escherichia coli* MST5 (KY550381). In terms of isolation source, *S. haemolyticus* MST1 was isolated from a traumatic wound, while *P. aeruginosa* MST2 and *E. coli* MST4 were procured from hernia surgical wounds, and *K. pneumoniae* MST3 and *E. coli* MST5 were obtained from diabetic foot ulcers. Antibiotic sensitivity tests exposed that *K. pneumoniae* MST3, *E. coli* MST4, and *E. coli* MST5 are extended-spectrum *β*-lactamases (ESBLs) bacteria. Moreover, *S. haemolyticus* MST1 belongs to the methicillin-resistant coagulase-negative *staphylococcus* (MRCoNS), whereas *P. aeruginosa* MST2 exhibited resistance to common empirical bactericidal antibiotics. Overall, the study provides new insights into the prevalent MDR bacteria in Egypt for further use as specific models in formulating antibacterial wound dressings.

## 1 Introduction

Skin functions as a crucial barrier against the environment and microbial invasions, performing a variety of critical defensive undertakings. However, once the integrity of the normal anatomical structure of the skin is endangered, the specific protection mechanism of the skin deteriorates, necessitating the application of extra protection to the wounded skin (Gruppuso et al., 2021, Tamer et al., 2021). The disruption of skin properties could be due to surgical procedures, chemical and physical or thermal actions (Maillard et al., 2021). The wound healing process of the wounded skin is greatly coordinated, with the recruitment of different dermal cells and active molecules to quickly close the injured area and restore the damaged tissues (Hassan et al., 2021, Yazarlu et al., 2021). Nevertheless, wound exudates put damaged skin at risk of colonization of pathogenic microorganisms, involving Gram-negative bacteria, such as *Pseudomonas aeruginosa* (*P. aeruginosa*), *Escherichia coli* (*E. coli*), *Acinetobacter baumannii* (*A. baumannii*), and *Klebsiella pneumoniae* (*K. pneumoniae*), Gram-positive bacteria, such as *Staphylococcus aureus* (*S. aureus*) and methicillin-resistant *S. au*reus (MRSA), and fungal strains, such as *Candida albicans* (*C. albicans*) (Hassan et al., 2019, Zhou et al., 2019). Undoubtedly, the incidence of pathogens in wound sites impedes wound healing and might even incite septicemia, which extremely menaces the life of patients (Puca et al., 2021). Thus, it is critical to diagnose the pathogens and select effective medicaments to thwart the infected injuries in clinical applications (Poletajew et al., 2021).

The conventional strategies for combating microbial infections for wound treatment are primarily based on antibiotics as the most efficient method. However, the extensive and uncontrolled use of broad-spectrum antimicrobial treatment by a clinician to manage an infection has a detrimental influence on populations of vulnerable microorganisms that are a typical component of normal microbial flora. Moreover, the abuse or overuse of antibiotics in hospitals generates multidrug-resistant (MDR) bacteria with continuous evolution and expansion responsible for nosocomial and community-acquired illnesses, posing a serious menace to human health and safety (Baym et al., 2016). Furthermore, MDR bacteria can insulate themselves against harsh environments, allowing them to persist and proliferate by adopting advantageous alterations (Baym et al., 2016).

**Fig. 1:**
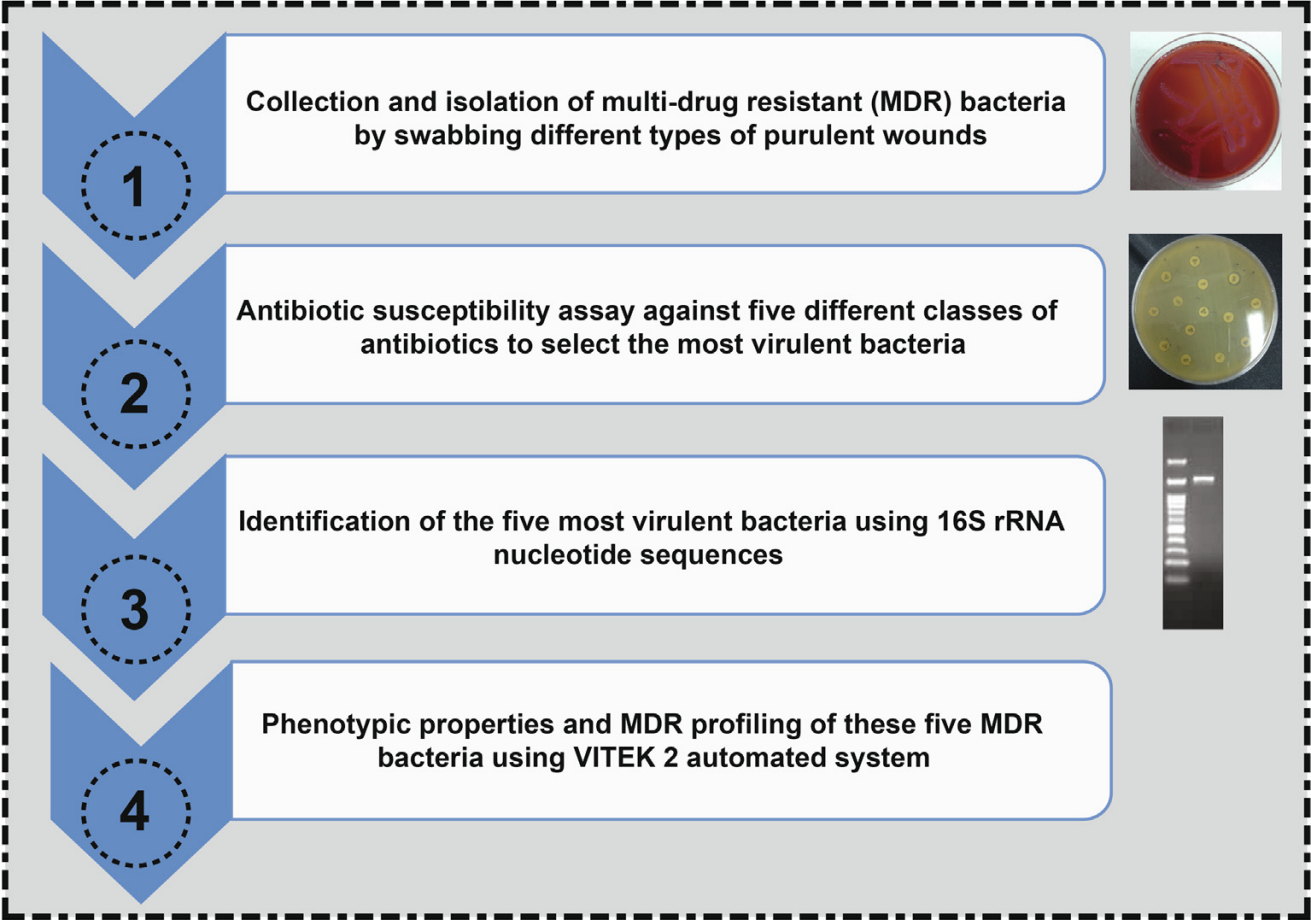
Schematic diagram exhibits the collection and isolation of MDR bacteria from different types of wounds and the procedures used to identify and characterize the most virulent bacteria.

Regardless of the kind of wound, wound infections are related to morbidity and death in patients in developing countries. Additionally, wound treatment failure burdens healthcare due to extended hospitalization and massive antibiotic dosage (Khan et al., 2017, Beyene et al., 2019). Therefore, it is of great significance to determine the microbial pathogens and antibiotic sensitivity patterns to implement an efficient therapeutic strategy for hindering the proliferation of pathogens without instigating side effects (Khan et al., 2017, Taati Moghadam et al., 2020). Furthermore, the World Health Organization has prioritized antimicrobial resistance in this group of bacteria as one of the most serious threats to global health. Therefore, it is encouraging research in this domain to curtail the mutations of bacteria by finding out the most effective antibiotic without delay (Sora et al., 2021). In this study, we sought to survey different types of wounds in hospitals in Alexandria, North Egypt, to detect and identify the dominant pathogenic bacteria associated with those infected wounds as portrayed in Fig. 1. We thus selected the most virulent bacteria for investigating their biochemical characteristics and antibiotic sensitivity patterns, adopting agar-disc diffusion as a conventional approach along with the VITEK 2 system. Moreover, the selected MDR bacteria were also identified using the 16S rRNA nucleotide sequence. Therefore, further investigations with more focus on the genes of those strains responsible for their resistance to antibiotics are suggested. Moreover, we would use these MDR bacteria as significant models to explore novel wound dressings to impede the growth of these pathogenic bacteria and accelerate wound healing, particularly some of those bacteria isolated from diabetic foot ulcers.

## 2 Materials and methods

### 2.1 Sampling collection and technique

The current study was conducted on 15 patients with different types of purulent wounds in Alexandria hospitals, North Egypt. The bacterial analyses were performed in the City of Scientific Research and Technological Applications, Alexandria, Egypt.

Prior to undertaking the investigations, informed consent was obtained from all patients after an explanation of the project in order to survey the frequency of multidrug-resistant bacteria used by the collected specimens. Wound samples were obtained and addressed in compliance with the Research Ethical Committee, which has been issued by the National Health and Medical Research Council Policies and the recommendations of the Ministry of Health and Population, High Committee of Medical Specialties, Egypt. Prior to swabbing the wounds, they were cleansed with sterilized saline solution (0.85%) and then swabbed by means of sterilized cotton swabs to isolate the most predominant pathogenic bacteria. As a result, the examined injuries were deeply infected, with purulent discharge and inflammatory symptoms. Upon collection, each sample was designated with the patient identifying number, date, and time of collection for future reference. Afterwards, the samples were immediately transferred to the microbiological lab under aseptic conditions.

### 2.2 Isolation of pathogenic bacteria

Each swab was spread over a Luria-Bertani (LB) agar plate alongside a MacConkey-Agar plate as a selective medium to define Gram-positive and Gram-negative bacteria. After 18 h of incubation at 37 °C, the separate colonies were then purified on LB agar (10 g tryptone, 5 g yeast extract, 10 g NaCl, and 15 g agar per 1 L of distilled water) and incubated under similar conditions. To evade spontaneous genetic mutations, the purified bacterial isolates were stored in glycerol at −80 °C for further use.

### 2.3 Antibacterial susceptibility evaluations

Antibiotic susceptibility assays of bacteria isolated from the previous step were conducted employing the disc diffusion method according to the procedure of Hassan et al., (2018). In addition, the turbidities of bacterial cultures were adapted in accordance with McFarland standards 0.5 in a sterilized LB medium by means of a spectrophotometer prior to culturing on LB agar plates. Finally, we picked nine antibiotic discs (Bioanalyse Co., Ltd., Ankara, Turkey), representing five classes of antibiotics, which substantially apply to frustrating bacteria in infected wound beds.

The antibiotics used in this experiment are classified into the following five classes, including their concentrations: (I) Penicillins/beta-lactamase inhibitors combinations: amoxicillin/clavulanic acid 30 µg (Am./Cl.), (II) Cephalosporins: 3rd generation including cefoperazone 75 µg (Cef.), (III) Quinolone antibiotics: 2nd generation including norfloxacin 10 µg (Norf.), and ofloxacin 5 µg (Ofl.), 3rd generation including levofloxacin 5 µg (Lev.), (IV) Penicillins: penicillin 10 µg (Peni) and ampicillin 10 µg (Amp.), (V) Aminoglycosides: streptomycin 10 µg (Strept.) and amikacin 30 µg (Ak.). The data were analyzed and reported based on the guidelines furnished by the manufacturer for the antibiotic sensitivity evaluations. Consequently, pathogenic isolated bacteria that could repel the action of at least seven tested antibiotics were considered the greatest resistant bacteria and selected for further examinations. In addition, the morphological features of bacterial isolates were probed following the morphology of colonies and microscopic examination.

### 2.4 Identification of virulent bacteria

#### 2.4.1 Molecular identification

The most virulent bacteria were identified by 16S rRNA nucleotide sequence approach. First, the 16S rRNA genes were amplified from the respective genomic DNA for each bacterial isolate by mean of a PCR thermal cycler utilizing 16S rRNA primers: 27F (5′-GAGAGTTTGATCCTGGCTCAG-3′) and 1541R (5′-AAGGAGGTGATCCAGCCGC-3′) as previously described by Hassan et al., (2020).

The PCR reactions were executed as follows: 5 min at 95 °C for the denaturation process, followed by 35 cycles of 1 min at 95 °C, 1 min at 56 °C, and 1.5 min at 72 °C, with a final extension of 10 min at 72 °C. In addition, the amplified 16S rRNA genes were probed on an agarose gel (1.5%) compared to a 100 bp DNA ladder (100–3000 bp), and snapshots of the gels were taken by means of a gel documentation system.

Each PCR fragment was sliced from the gel and then cleaned up using the QIAquick Gel Extraction Kit (Qiagen, USA) before being sent to Sigma Company for the nucleotide sequence analysis according to the procedures for the enzymatic chain terminator approach.

Subsequently, the nucleotide sequence of each 16S rRNA gene was analyzed by means of the Basic Local Alignment Search Tool for nucleotides (BLAST) in relation to the corresponding nucleotide sequences accessible in the National Center for Biotechnology Information GenBank (NCBI GenBank). These homologous nucleotide sequences were further used for the alignment analysis employing Clustal W in MEGA7 software, and phylogenetic trees were designed following the neighbour-joining tree with bootstrap values of 500 (El-Fakharany et al., 2016, Kumar et al., 2016, Abol-Fotouh et al., 2021).

#### 2.4.2 Phenotypic and antibiotic sensitivity properties by VITEK 2 system

The molecular identification was verified by means of the VITEK 2 automated system to phenotypically characterize the bacterial isolates. Gram-negative and Gram-positive bacteria were characterized using specific GN and GP cards, respectively, integrated into Biomerieux VITEK 2. The pathogenic bacteria were grown for 16 h at 37 °C in the nutrient broth. The turbidities of the bacterial cultures were then diluted and adapted to McFarland standards of 0.5 as previously described. Finally, the cell suspensions were placed onto the respective cards; hence, the biochemical and antibiotic sensitivity characteristics were explored.

## 3 Results

### 3.1 Wound swabs and culture analysis

**Fig. 2:**
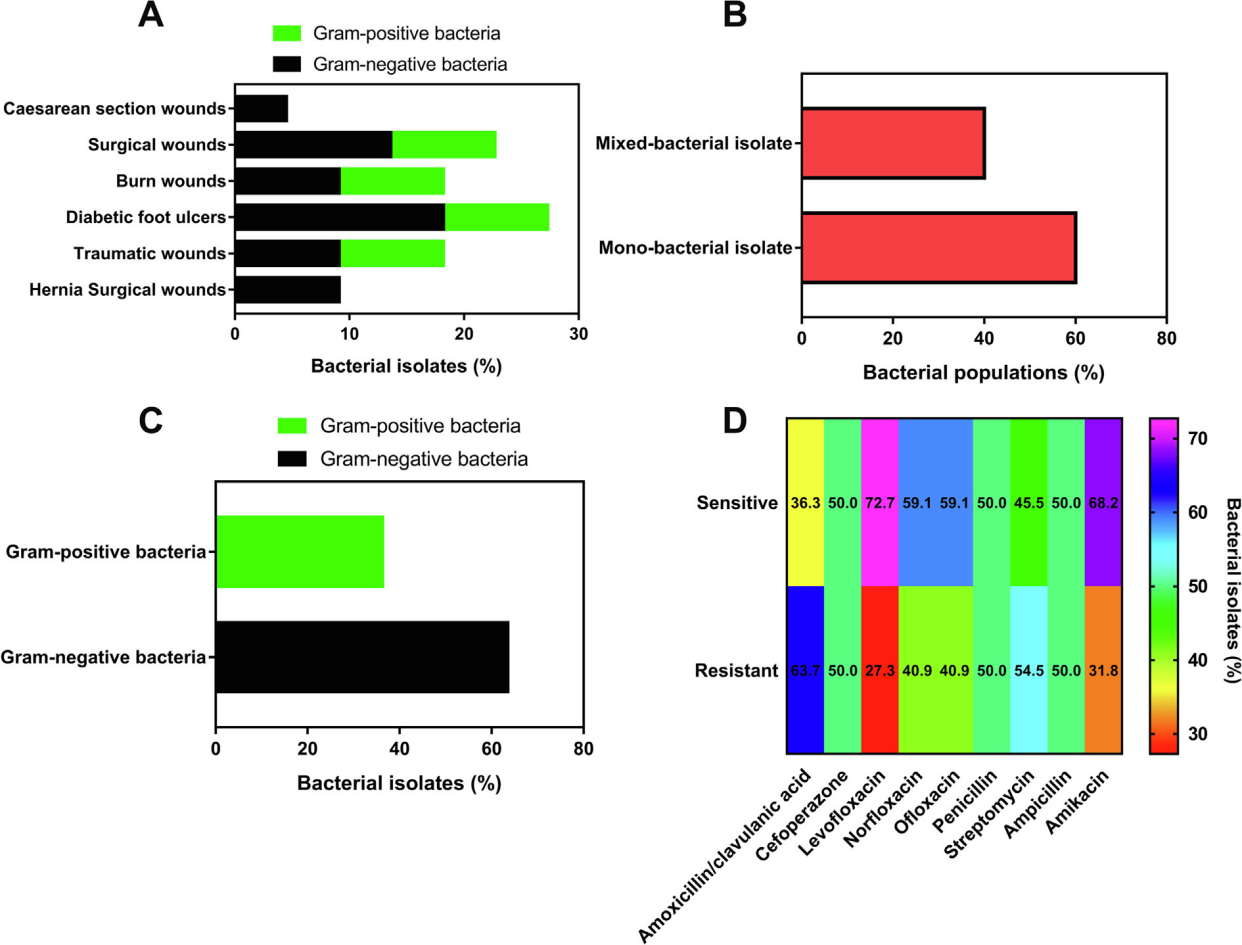
(A) Bacterial isolates from different types of wounded skin, showing the incidence of Gram-negative and Gram-positive bacteria isolated from each type of wound. (B) Bacterial populations reveal the dominance of mono-bacterial infection throughout the collected samples. (C) Percentage of Gram-negative and Gram-positive bacteria obtained from the entire specimens, exhibiting the preponderance of Gram-negative bacteria. (D) Antibiotic susceptibility patterns of the MDR bacteria isolated from the infected wounds using the agar-disc diffusion method in relation to nine antibiotics, which belong to five classes of antibiotics with different mechanisms against pathogenic bacteria.

In this research, MDR bacteria, including Gram-positive and Gram-negative bacteria, were isolated from various inflamed wounds as presented in Fig. 2A. In addition, a set of fifteen swabs were considered from debilitated wounds with significant secretions of purulence, yielding twenty-two pathogenic bacteria. The highest percentage of bacterial isolates emerged in diabetic foot ulcers with the prevalence of Gram-negative bacteria. From the infected wounds, one or poly-bacterial isolates possessed virulent bacteria, which could give rise to medical burdens were obtained.

From the data in Fig. 2B and Table 1, it can be extrapolated that only one bacterial isolate could be obtained from 60% of the entire swabs. By contrast, the episode of mixed pathogenic bacteria was observed in 40%, including one swab with triple bacterial isolates from a burn wound, representing 6.7% and 16.6% of the whole swabs and poly-infected injuries, respectively. Furthermore, Fig. 2C and Table 1 exhibit the preponderance of Gram-negative bacteria in the examined wounds, recording 63.6%, while the rest of the pathogenic bacteria (36.4%) belonged to Gram-positive bacteria.

**Table 1:** Antibiotic susceptibility patterns of MDR bacteria isolated from different types of wounds infected with pathogenic bacteria.

**Bacteria No.**	**Patients’ number and wound type**	**Type of bacteria**	**Antibiotic susceptibility pattern**	
			**Sensitive**	**Resistant**
1	(1) Hernia surgical wound	G-ve	Ak.	Am. /CL., Cef., Lev., Norf., Ofl., Peni.,Strept., Amp.
2	(1) Hernia surgical wound	G-ve		Am./Cl., Cef., Lev., Norf., Ofl., Peni., Strept., Amp., Ak.
3	(2) Traumatic wound	G+ve	Am./Cl., Norf., Peni., Amp., Ak.	Cef., Lev., Ofl., Strept.
4	(3) Burn wound	G-ve	Lev., Ofl., Strept., Amp., Ak.	Am./Cl.,Cef., Norf., Peni.
5	(4) Traumatic wound	G-ve	Am./Cl., Cef., Lev., Norf., Ofl., Amp.	Peni., Strept. Ak.
6	(5) Caesarean section wound	G-ve	Lev., Ofl., Peni., Strept., Amp.,Ak.	Am./Cl., Cef., Norf.
7	(6) Surgical wound	G-ve	Cef., Lev., Norf., Ofl., Peni., Amp., Ak.	Am./Cl.,Strept.
8	(7) Surgical wound	G+ve	Cef., Lev., Norf., Amp., Ak.	Am./Cl., Ofl., Peni., Strept.
9	(8) Diabetic foot ulcer	G-ve	Ak.	Am./Cl., Cef., Lev., Norf., Ofl., Peni., Strept., Amp.
10	(9) Diabetic foot ulcer	G-ve	Lev., Norf.	Am./Cl., Cef., Ofl., Peni., Strept., Amp., Ak.
11	(9) Diabetic foot ulcer	G+ve	Am./Cl., Cef., Lev., Ofl., Strept., Amp., Ak.	Norf., Peni.
12	(10) Surgical wound	G-ve	Lev., Norf., Peni., Ak.	Am./Cl., Cef., Ofl., Strept., Amp.
13	(11) Traumatic wound	G+ve	Norf.	Am./Cl., Cef., Lev., Ofl., Peni., Strept., Amp., Ak.
14	(11) Traumatic wound	G-ve	Cef., Lev., Norf., Peni., Strept.	Am./Cl., Ofl., Amp., Ak.
15	(12) Surgical wound	G-ve	Cef., Lev., Norf., Ofl. Peni., Strept.,Amp.	Am./Cl., Ak.
16	(12) Surgical wound	G+ve	Cef., Lev., Norf., Ofl., Peni., Amp.	Am./Cl., Strep., Ak.
17	(13) Diabetic foot ulcer	G-ve	Am./Cl., Cef., Lev., Norf., Strept., Ak.	Ofl., Peni., Amp.
18	(14) Burn wound	G+ve	Am./Cl., Cef., Lev., Norf., Peni., Strept., Ak.	Ofl., Amp.
19	(14) Burn wound	G-ve	Am./Cl., Lev., Peni., Strept., Ak.	Cef., Norf., Ofl., Amp.
20	(14) Burn wound	G+ve	Am./Cl., Norf., Ofl., Peni., Amp., Ak.	Cef., Lev., Strept.
21	(15) Diabetic foot ulcer	G-ve	Am./Cl., Cef., Lev., Ofl., Strept., Amp., Ak.	Norf., Peni.
22	(15) Diabetic foot ulcer	G+ve	Cef., Lev., Ofl., Peni., Strept., Ak.	Am./Cl., Norf., Amp.

Am./Cl. (Amoxicillin/clavulanic acid) (30 µg), Cef. (Cefoperazone) (75 µg), Lev. (Levofloxacin) (5 µg), Norf. (Norfloxacin) (10 µg), Ofl. (Ofloxacin) (5 µg), Peni. (Penicillin) (10 µg), Strept. (Streptomycin) (10 µg), Amp. (Ampicillin) (10 µg), Ak. (Amikacin) (30 µg).

### 3.2 Antibiotic sensitivity patterns

In order to determine the most infectious bacteria, we performed an antibiotic disk diffusion approach, adopting different antibiotics from various classes, which vary according to their mechanism against the tested bacteria. It could be extrapolated from Fig. 2D and Table 1 that amikacin thwarted the bacterial growth of 68.2% with regards to the total bacterial isolates. Besides, the major bacteria affected by amikacin were Gram-negative, up to 64.3% of the entire Gram-negative bacteria. On the other hand, levofloxacin showed the maximum growth inhibition rate against the tested bacteria reached 72.7%, while amoxicillin/clavulanic acid exerted a minimum growth inhibition ratio of 36.3%. Additionally, three antibiotics, including cefoperazone, penicillin, and ampicillin, impeded the bacterial growth of 50% of the bacteria. However, penicillin and ampicillin have identical modes of action against bacteria since they are derived from the penicillins class of antibiotics.

Moreover, it can be seen that the frequency of resistant bacteria to streptomycin was 54.5%, whereas pathogenic bacteria with a ratio of 40.9% counteracted the activity of ofloxacin and norfloxacin. It could be deduced from these findings that the majority of these pathogenic bacteria could withstand at least four antibiotics with unrelated mechanisms, demonstrating the virulence of those bacteria as MDR. However, the most apparent findings to emerge from the antibiotic sensitivity assay are that the isolated bacteria nos. 1, 9, 10, and 13 showed considerable resistance to the most studied antibiotics. In contrast, bacterial isolate no. 2 revealed insusceptibility in relation to the entire antibiotic discs. Precisely, bacterial isolates nos. 1 and 9 repelled the antibacterial performance of the whole antibiotics except amikacin. Furthermore, norfloxacin revealed remarkable antibacterial capacities against strains nos. 10, and 13. Based on the types of wounds from which each bacterial isolate was obtained, bacterial isolates nos. 1 and 2 were obtained from hernia surgical wounds, whereas bacterial isolates nos. 9 and10 were isolated from diabetic foot ulcers, and bacterial isolate no. 13 was isolated from a traumatic wound.

Taken together, five virulent bacterial isolates displayed superior resistance to almost all antibiotic discs; thus, they were identified following their molecular and phenotypic properties by the 16S rRNA sequencing approach and the VITEK 2 system, respectively. Accordingly, we labeled the bacterial isolates nos. 1, 2, 9, 10, and 13 as MST4, MST2, MST3, MST5, and MST1, respectively.

### 3.3 Identification of the most infectious bacteria

**Fig. 3:**
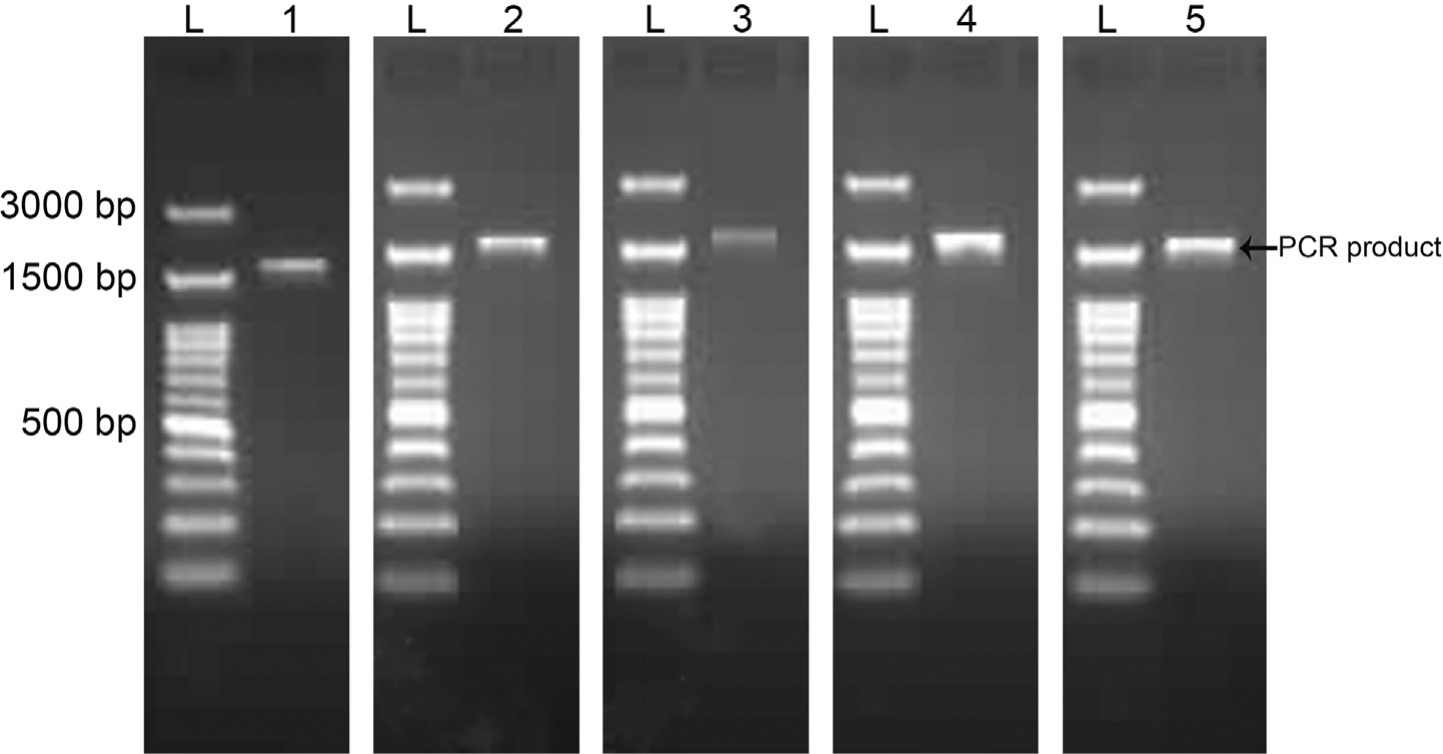
Amplified 16S rRNA genes with sizes of approximately 1500 bp from the genomic DNA of *S. haemolyticus* MST1, *P. aeruginosa* MST2, *K. pneumoniae* MST3, *E. coli* MST4, and *E. coli* MST5.

With the use of a molecular method following the nucleotide sequences of the 16S rRNA gene, we identified the bacterial isolates MST1, MST2, MST3, MST4, and MST5. As shown in Fig. 3, fragments with a size of approximately 1.5 kb of 16S rRNA genes were obtained.

**Fig. 4:**
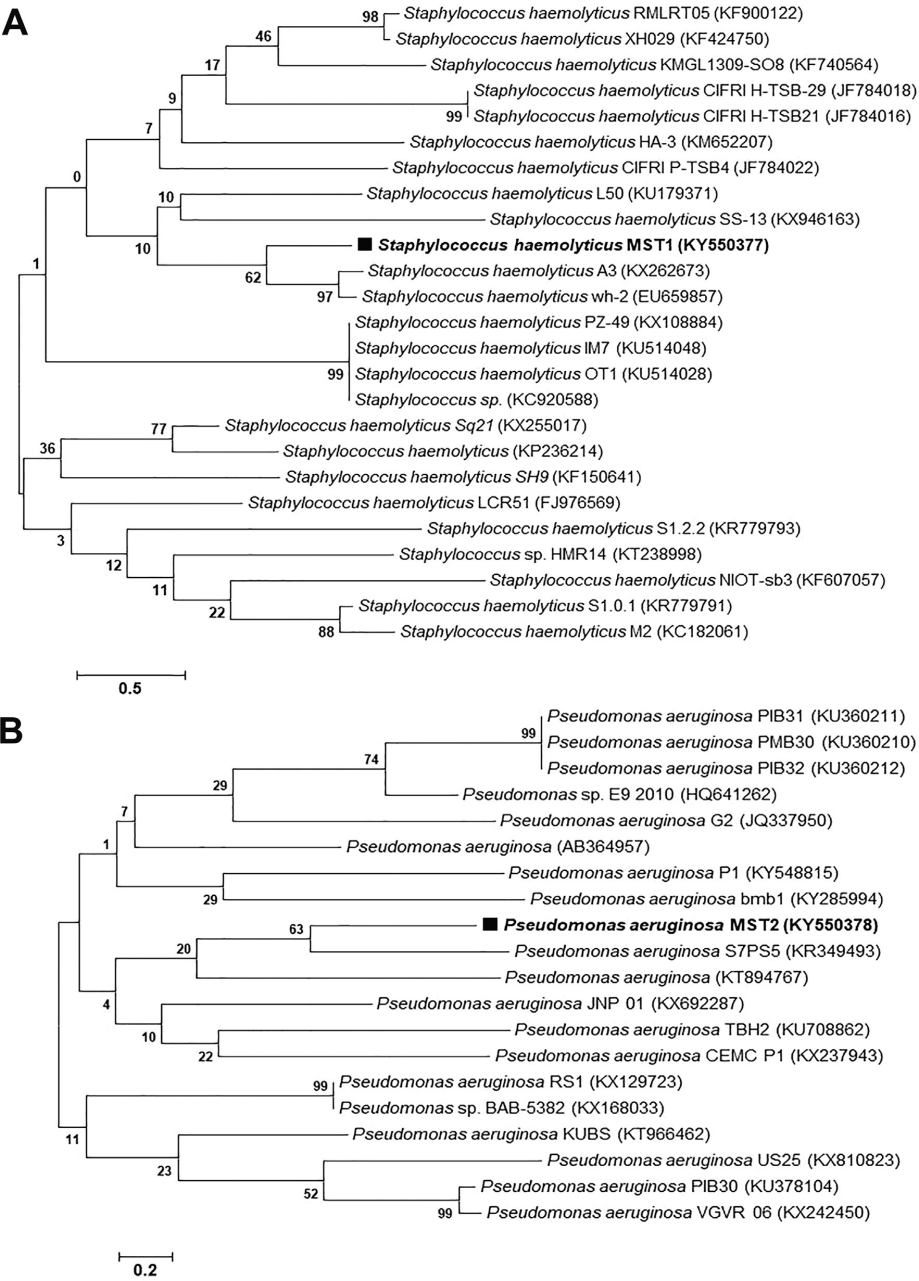
Phylogenetic trees of (A) *S. haemolyticus* MST1 (KY550377) and (B) *P. aeruginosa* MST2 (KY550378) reveal their evolutionary relationships with regards to the closest bacteria on the basis of 16S rRNA nucleotide sequences retrieved from the GenBank database, and accession numbers of the sequences are indicated in parentheses. The phylogenetic trees were constructed employing the Neighbor-Joining tree with bootstrap values of 500 replicates, and the bars denote Jukes-Cantor distances of 0.5 and 0.2 for *S. haemolyticus* MST1 and *P. aeruginosa* MST2, respectively.

**Fig. 5:**
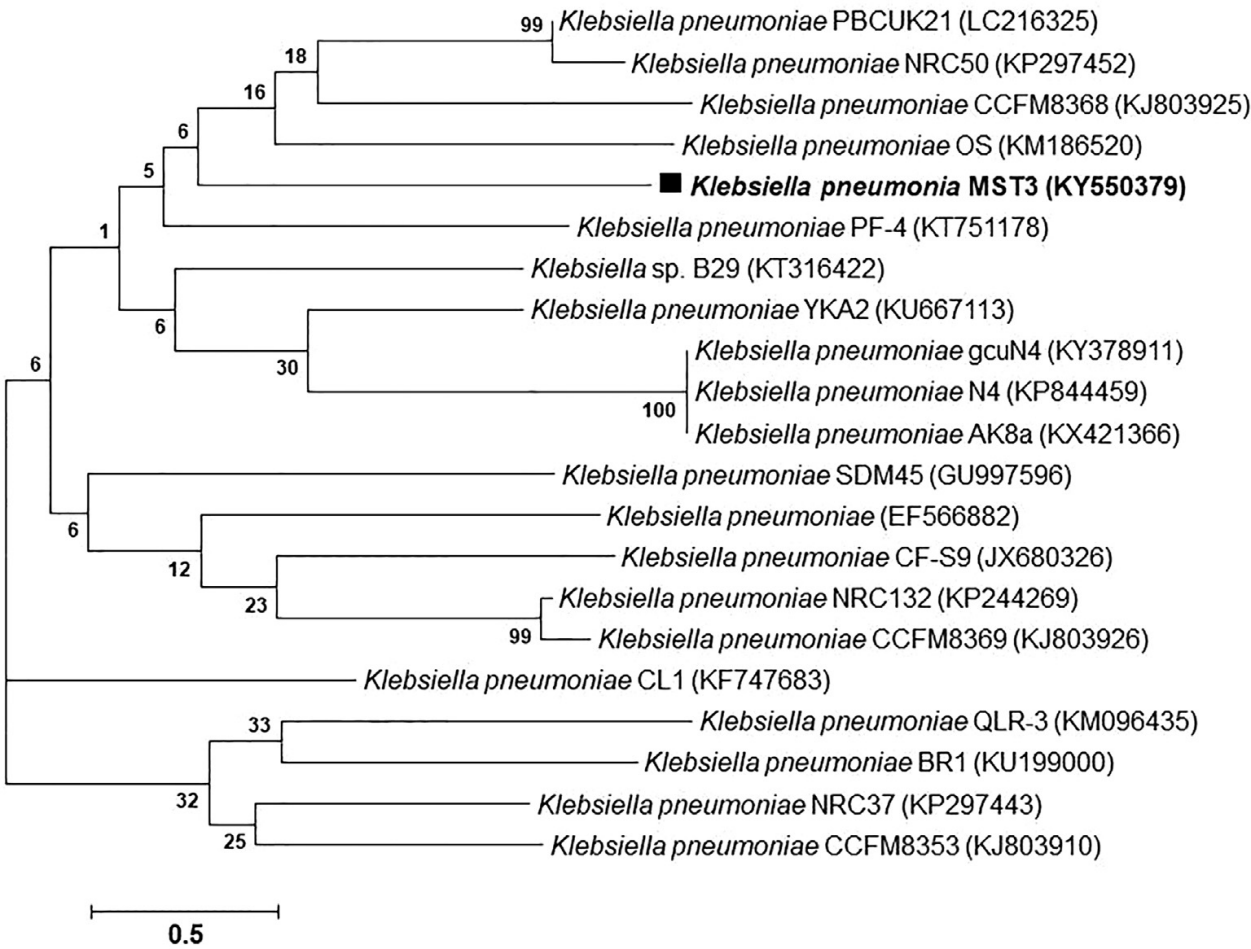
A phylogenetic tree of *K. pneumoniae* MST3 (KY550379) reveals its evolutionary relationships to the closest bacteria based on 16S rRNA nucleotide sequences retrieved from the GenBank database, with accession numbers of the sequences indicated in parentheses. The Phylogenetic tree was constructed by employing the Neighbor-Joining tree with bootstrap values of 500 replicates, and a bar denotes the Jukes-Cantor distance of 0.5**.**

**Fig. 6:**
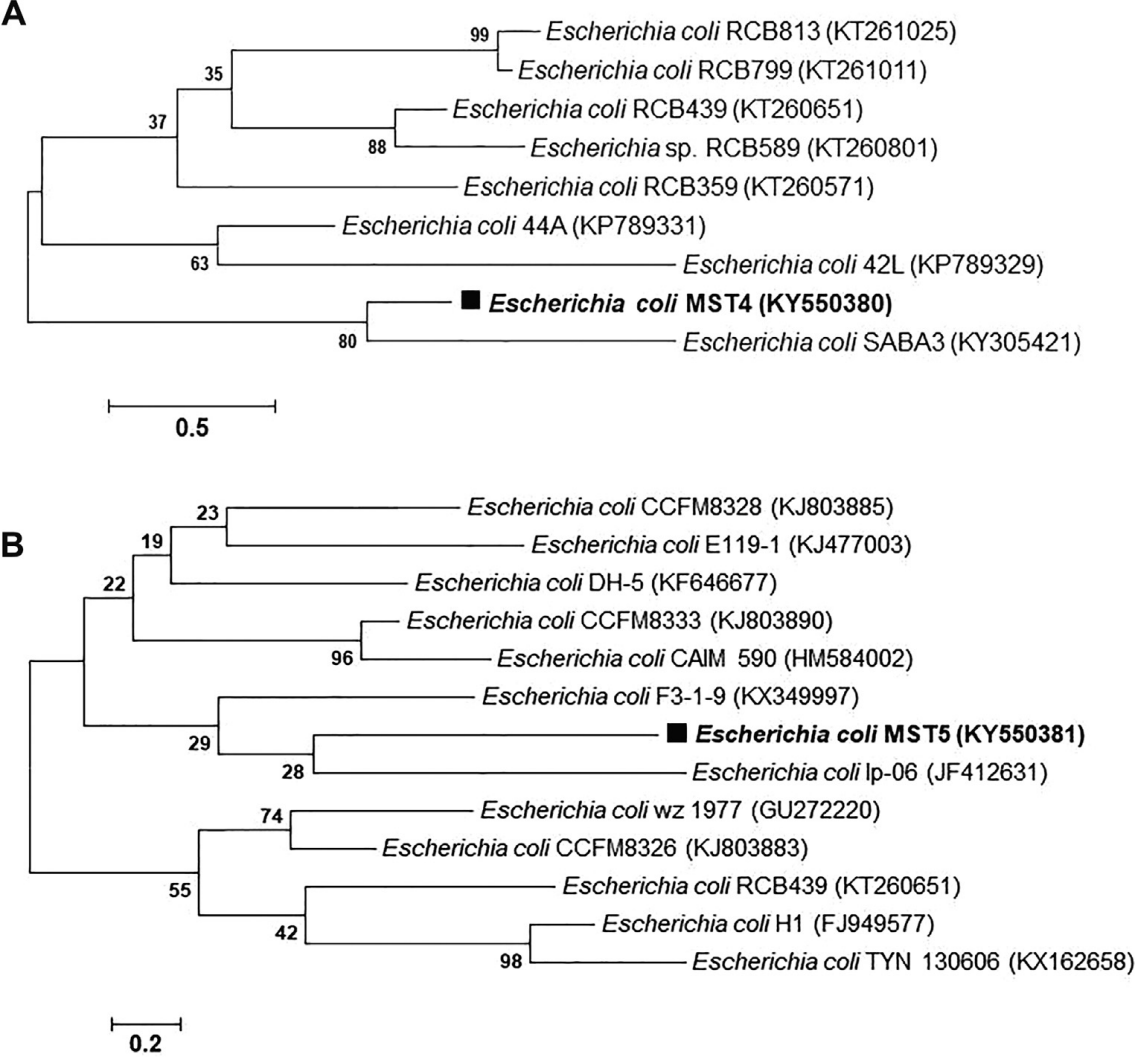
Phylogenetic trees of (A) *E. coli* MST4 (KY550380) and (B) *E. coli* MST5 (KY550381) reveal their evolutionary relationships with regards to the closest bacteria on the basis of 16S rRNA nucleotide sequences retrieved from the GenBank database, and accession numbers of the sequences are indicated in parentheses. The phylogenetic trees were constructed employing the Neighbor-Joining tree with bootstrap values of 500 replicates, and the bars denote Jukes-Cantor distances of 0.5 and 0.2 for *E. coli* MST4 and *E. coli* MST5, respectively.

The obtained 16S rRNA nucleotide sequences of 750 bp, 1458 bp, 1217 bp, 1482 bp, and 1459 bp for bacterial isolates MST1, MST2, MST3, MST4, and MST5, respectively, were deposited in the GenBank database after analysis using BLASTn. The alignment results showed the similarity of 16S rRNA nucleotide sequences from isolates MST1, MST2, MST3, MST4, and MST5 with percentages of 95% with *Staphylococcus haemolyticus*, 98% with *Pseudomonas aeruginosa*, 95% with *Klebsiella pneumoniae*, 99% with *Escherichia coli*, and 99% with *Escherichia coli*, respectively. The 16S rRNA nucleotide sequences of the identified isolates were deposited in GenBank with specified accession numbers as follows: *Staphylococcus haemolyticus* MST1 (KY550377), *Pseudomonas aeruginosa* MST2 (KY550378), *Klebsiella pneumoniae* MST3 (KY550379), *Escherichia coli* MST4 (KY550380), and *Escherichia coli* MST5 (KY550381). Fig. 4, Fig. 5, Fig. 6 illustrate the phylogenetic trees of the bacterial strains on the basis of the nucleotide sequences in comparison with the homologous nucleotide sequences retrieved from GenBank, indicating the position of each strain within the closest strains.

The VITEK 2 system confirmed the molecular identification of the bacterial strains as given in Table 2. During the identification analysis, various biochemical tests of the bacterial strains were studied, including catalase, coagulase, oxidase, sugar fermentation, enzyme activities, and the influence of some inhibitory compounds. Furthermore, the antibiotic susceptibility patterns of the strains were explored using different antibiotics as presented in Table 3, showing the high resistance of these MDR bacteria. Taken together, the MDR bacterial strains MST1, MST2, MST3, MST4, and MST5 are *S. haemolyticus* MST1, *P. aeruginosa* MST2, *K. pneumoniae* MST3, *E. coli* MST4, and *E. coli* MST5.

**Table 2:** Phenotypic properties of *S. haemolyticus* MST1, *P. aeruginosa* MST2, *K. pneumoniae* MST3, *E. coli* MST4, and *E. coli* MST5 using VITEK 2.

	Test	*S. haemolyticus* MST1	Test	*P. aeruginosa*MST2	*K. pneumoniae* MST3	*E. coli* MST4	*E. coli* MST5
1	Ala-phe-pro-arylamidase	–	Ala-phe-pro-arylamidase	–	–	–	–
2	Alpha-mannosidase	–	Adonitol	–	+	–	–
3	L-Pyrrolydonyl-arylamidase	+	L-Pyrrolydonyl-arylamidase	–	+	–	–
4	LeucineArylamidase	–	L-Arabitol	–	–	–	–
5	Beta-glucuronidase	–	D-Cellobiose‘	–	+	–	–
6	Beta-galactosidase	–	Beta-galactosidase	–	+	+	+
7	D-Amygdalin	–	H_2_S production	–	–	–	–
8	Phosphatidylinositol Phospholipase C	–	Beta-n-acetyl glucosaminidase	–	–	–	–
9	D-Xylose	–	Glutamylarylamidasepna	–	–	–	–
10	Arginine Dihydrolase 1	+	D-glucose	+	+	+	+
11	Cyclodextrin	–	Gamma-glutamyl-transferase	+	+	–	–
12	L-Aspartate Arylamidase	–	Fermentation/ glucose	–	+	+	+
13	Beta Galactopyranosidase	–	Beta-glucosidase	–	+	–	–
14	D-Maltose	+	D-Maltose	–	+	+	+
15	D-mannitol	+	D-mannitol	+	+	+	+
16	D-mannose	–	D-mannose	+	+	+	+
17	Methyl-B-D-Glucoyranosidase	–	Beta-xylosidase	–	–	–	–
18	Pullulan	–	Beta-alanine arylamidasepna	+	–	–	–
19	L-prolinearylamidase	–	L-proline arylamidase	+	+	–	–
20	Lactose	+	Lipase	–	–	–	–
21	N-acetyl-D-Glucosamine	+	Palatinose	–	+	–	–
22	Tyrosine arylamidase	–	Tyrosine arylamidase	+	+	+	–
23	Urease	–	Urease	–	+	–	–
24	D-sorbitol	–	D-sorbitol	–	+	+	+
25	Saccharose/Sucrose	+	Saccharose/sucrose	–	+	+	+
26	Salicin	–	D-tagatose	–	–	–	–
27	D-trehalose	+	D-trehalose	–	+	+	+
28	Bacitracin Resistance	+	Citrate (sodium)	+	+	–	–
29	Novobiocin	–	Malonate	+	+	–	–
30	Growth In 6.5% NaCl	+	5-Keto-d-gluconate	–	–	–	–
31	L-lactate alkalinisation	+	L-lactate alkalinisation	+	+	+	–
32	Alpha-glucosidase	+	Alpha-glucosidase	–	–	–	–
33	Arginine Dihydrolase 2	–	Succinate alkalinisation	+	+	+	–
34	Optochin Resistance	+	Beta-n-acetyl-galactosaminidase	–	–	–	–
35	Alpha-galactosidase	–	Alpha-galactosidase	–	+	+	–
36	Phosphatase	–	Phosphatase	–	+	–	–
37	Polymixin B Resistance	–	Glycine arylamidase	–	+	–	–
38	D-Galactose	+	Ornithine decarboxylase	–	–	–	+
39	D-Ribose	+	Lysine decarboxylase	–	+	+	+
40	Alanine Arylamidase	–	L-histidine assimilation	+	–	–	–
41	D-Raffinose	–	Coumarate	+	–	+	+
42	Beta-glucoronidase	–	Beta-glucoronidase	–	–	+	+
43	O/1 29 resistance (comp.vibrio.)	+	O/1 29 resistance (comp.vibrio.)	+	+	+	+

**Table 3:** Antibiotic susceptibility patterns of *S. haemolyticus* MST1, *P. aeruginosa* MST2, *K. pneumoniae* MST3, *E. coli* MST4, and *E. coli* MST5 using VITEK 2.

Antibiotic	**Interpretation**				
	*S. haemolyticus* MST1	*P. aeruginosa* MST2	*K. pneumoniae* MST3	*E. coli* MST4	*E. coli* MST5
Cefoxitin screen	+ve	–	–	–	–
Benzylpenicillin	R	–	–	–	–
Oxacillin	R	–	–	–	–
Imipenem	R	*I	R	S	S
Gentamicin	R	–	–	–	–
Ciprofloxacin	R	–	–	–	–
Moxifloxacin	I	–	–	–	–
Inducible Clindamycin Resistance	-ve	–	–	–	–
Erythromycin	R	–	–	–	–
Clindamycin	S	–	–	–	–
Linezolid	S	–	–	–	–
Teicoplanin	S	–	–	–	–
Vancomycin	S	–	–	–	–
Tetracycline	S	–	–	–	–
Tigecycline	S	–	–	–	–
Fosfomycin	R	–	–	–	–
Fusidic Acid	R	–	–	–	–
Rifampicin	S	–	–	–	–
Trimethoprim/Sulfamethoxazole	R	R	R	R	R
Ticarcillin	–	R	–	–	–
Ticarcillin/Clavulanic Acid	–	R	–	–	–
Piperacillin	–	R	–	–	–
Ceftazidime	–	I	–	–	–
Pefloxacin	–	R	–	–	–
Minocycline	–	R	–	–	–
Colistin	–	S	–	–	–
Meropenem	–	I	S	S	S
Amikacin	–	R	S	S	S
Gentamicin	–	R	R	R	S
Tobramycin	–	S	R	R	S
Ciprofloxacin	–	R	I	R	S
Cefepime	–	S	*R	*R	*R
ESBL	–	–	+ve	+ve	+ve
Ampicillin	–	–	R	R	R
Ampicillin / Sulbactam	–	–	R	R	S
+ Cefotaxime	–	–	R	R	R
Ceftriaxone	–	–	R	R	R
Cefazolin	–	–	R	R	R
Aztreonam	–	–	R	*R	R
Moxifloxacin	–	–	S	R	S
Ertapenem	–	–	R	S	S
Nitrofurantoin	–	–	S	I	S
Tigecycline	–	–	R	S	S

+ve = Deduced drug; * =AES modified; R = Resistant; S = Sensitive; I = Intermediate; - = not applicable.

## 4 Discussion

Wound infection is increasingly recognized as a serious, worldwide public health concern. It is associated with an increased risk of disease and morbidity for both patients. Specifically, for the patient, it provokes pain, discomfort, inconvenience, disability, financial drain, and even death owing to complications, such as septicemia. Furthermore, wound infection may financially strain healthcare systems due to the high cost of patient hospitalization and microbial infection control. Pathogenic microorganisms engender wound infection; however, pathogenic bacteria are the most predominant at the sites of inflamed wounds. Thus, despite increasing concerns about antibiotic-resistant bacteria, the use of conventional antibiotics is still recommended to stall these kinds of infections (Bessa et al., 2015).

Nevertheless, using these empirical antibiotics gave rise to the emergence of MDR bacteria, predisposing patients to adverse influences and even death in some cases. Furthermore, wounds furnish an encouraging environment for microorganism harboring, which hinder the wound healing process (Canal et al., 2009). Frequent microorganisms colonizing wounds emanate from either the endogenous skin flora of patients or could be transmitted as cross-infection from the hospital surroundings. The last type tends to be more resistant to antibiotics than those developed out of endogenous skin flora (Baym et al., 2016). Thus, several bacterial pathogens correlated with epidemics of human disease have transformed into MDR organisms due to abuse or overuse of antibiotics (Hassan et al., 2019, Maillard et al., 2021). It is thus crucial to sustain antimicrobial agents' activity as long as possible during their application to prevent the unexpected mutations of pathogenic microorganisms (Chang et al., 2015).

In the current research, we surveyed the populations of MDR bacteria in different types of wounds treated with antibiotics in Alexandria, North Egypt. Following determinations of the most virulent bacteria, future investigations should seek to explore specific wound dressings to hamper such bacterial infections and ameliorate wound healing and skin rejuvenation.

Wound infections can be monomicrobial or polymicrobial depending on the state of the wounds and the patients' previous treatment; thus, different wounds infected with microorganisms do not have comparable populations and numbers of microorganisms (Upreti et al., 2018). For instance, the lack of nutrients and moisture in the skin are pivotal factors in diminishing the proliferation of fastidious bacteria, thereby reducing the incidence of Gram-positive bacteria (Cogen et al., 2008). In this research, the monobacterial infections of wounds exhibited a prevalence of 60% compared to 40% for polybacterial infections. These results are in agreement with those obtained by Asres et al., (2017), who showed monomicrobial and polymicrobial infection rates of 88.6% and 11.4%, respectively. Additionally, this also accords with earlier observations, which reported 72.8% for monomicrobial and 27.2% for polymicrobial infections (Bessa et al., 2015). In like manner, our findings match those observed in previous investigations, which found the predominance of monomicrobial infections (Kaftandzieva et al., 2012, Sánchez-Sánchez et al., 2017).

Our findings also revealed that Gram-negative bacteria were dominant in this survey with a ratio of 63.6%, whereas Gram-positive bacteria showed 36.4%. These findings are in line with those perceived in prior investigations, which displayed 71.6% for Gram-positive and 28.4% for Gram-negative (Upreti et al., 2018). Likewise, a prior study demonstrated that Gram-negative bacilli (70%) are more dominant than Gram-positive bacteria (30%) (Thanni et al., 2003). Besides, in another study, Gram-negative rods were the predominant and leading cause of wound infections and these outcomes agreed with previous studies in Asia and other African locations (Kassam et al., 2017, Yang et al., 2017, Moges et al., 2019).

In contrast, other investigations reported approximately similar percentages of Gram-positive and negative bacteria (Bessa et al., 2015, Asres et al., 2017). These differences are most likely due to variations in common nosocomial pathogens found in diverse hospital settings. The diversity of MDR bacteria in inflamed wounds might be explained by a number of variables, including demographics, age differences, gender, hospitalization length, and prior antibiotic treatment. Furthermore, hospitalization may substantially impact the prevalence and kind of MDR bacteria since patients are at risk of cross-infection with nosocomial infections that withstand some prescription antibiotics.

Beta-lactam antibiotics are the most commonly used antibiotics against pathogenic bacteria and are the leading source of *β*-lactam antibiotic-resistant bacteria in Gram-negative bacteria globally (Rodríguez et al., 2020). Consistent administration of *β*-lactam antibiotics and their interaction with bacteria has engendered the dynamic and continual development of *β*-lactamases, increasing their resistance even against recently formulated *β*-lactam antibiotics (Castanheira et al., 2021). These lactamases, extended-spectrum *β-*lactamases (ESBLs), are specific enzymes which have the competency to hydrolyze the penicillins group and other relevant antibiotics implicated *β-*lactam ring. Therefore, the emergence of ESBLs reduced the use of some of the newly developed antibiotics based on a *β-*lactam ring, such as cephalosporins and monobactams. Even though the frequency of ESBL strains is not entirely determined, they are obviously growing, with 10–40% of *E. coli* and *K. pneumoniae* menacing human beings (Shaikh et al., 2015). Therefore, the VITEK 2 automated system (bioMérieux) is broadly applied in clinical microbiology labs for repaid identification of pathogenic bacteria along with their antibiotic susceptibility profiling, particularly for detecting extended-spectrum *β*-lactamases (ESBLs) bacteria (Espinar et al., 2011).

In this study, the phenotypic analyses of the five pathogenic bacteria employing VITEK 2 AES manifested the resistance of three bacteria (*K. pneumoniae* MST3, *E. coli* MST4, and *E. coli* MST5) against ampicillin, cefotaxime, cefepime, ceftizoxime, cefazolin, and aztreonam, demonstrating that they are ESBL-producing bacteria. Although isolates MST4 and MST5 are both *E. coli*, they differ in their antibiotic susceptibility patterns, in which *E. coli* MST4 is more antibiotic resistant than *E. coli* MST5. Specifically, *E. coli* MST4 exposed resistance to comparable antibiotics like *E. coli* MST5, along with other common antibiotics used in hospitals, such as gentamicin, tobramycin, ciprofloxacin, and moxifloxacin. These differences could be primarily attributed to the types of wounds from which each strain was isolated along with the prior antibiotic scenario; in particular, *E. coli* MST4 was isolated from a hernia surgical wound, whereas *E. coli* MST5 was isolated from a diabetic foot ulcer. Recent investigations reported the isolation of ESBL-producing *E. coli* from community-acquired urinary tract infections (Jia et al., 2021).

On the other hand, the antibiotic susceptibility results exposed the substantial resistance of *K. pneumoniae* MST3 against most examined antibiotics. The critical issue is due to the prevalence of this species in most hospitals as a consequence of incessant mutations, and our strain was isolated from a diabetic foot ulcer. Therefore, the growth and rapid proliferation of such pathogenic bacteria should be hampered by using appropriate antimicrobial drugs earlier (Tommasi et al., 2015, Li et al., 2020).

With regard to *S. haemolyticus* MST1 and *P. aeruginosa* MST2, they were procured from traumatic and hernia surgical wounds, respectively. Considering *S. haemolyticus* MST1, it could stall the action of cefoxitin and oxacillin, pointing out that it can be categorized as methicillin-resistant coagulase-negative *staphylococcus* (MRCoNS) and *β-*lactam resistant bacteria since oxacillin is an antibiotic that belongs to the class of isoxazolyl penicillins (Bonvegna et al., 2021, Sonola et al., 2021). Besides, the insusceptibility of *S. haemolyticus* MST1 towards benzylpenicillin, oxacillin, imipenem, and other antibiotics such as gentamicin, ciprofloxacin, confirms these outcomes (Lowy, 2003, John et al.,2009). In addition, *P. aeruginosa* MST2 presented resistance to several antibiotics, including ticarcillin, piperacillin, ceftazidime (*β*-lactam antibiotics), and amikacin and gentamicin, which are commonly applied as rapid bactericidal antibiotics. This performance reflects the extremely deleterious influence of this strain on the patients. Critically, the World Health Organization classified methicillin-resistant coagulase-negative *staphylococcus* and *P. aeruginosa* as highly dangerous nosocomial pathogens, necessitating significant attention to developing novel antibacterial medicines (Lu et al., 2021). Additionally, given that MRSA and *P. aeruginosa* are aggressive biofilm producers, developing microbial biofilms with low penetrability and a sluggish metabolic rate of the encapsulated bacteria makes antibiotic treatments terribly resistant (Olsen, 2015, Lu et al., 2021).

Collectively, future studies on the current topic are therefore recommended to develop novel wound dressings with particular traits or drugs in order to effectively frustrate these MDR bacteria as reliable models for the incident pathogenic bacteria to promote the healing of injuries infected with MDR bacteria.

## 5 Conclusion

In this research, different MDR bacteria sheltered in different types of purulent wounds were isolated. We ascertained twenty-two bacteria as MDR bacterial strains due to their resistance to various classes of antibiotics. Among the total specimens, mono-bacterial infections and Gram-negative bacteria appeared to predominate. The most infectious bacteria, including five bacterial species, which frustrate the action of the major applied antibiotics, were characterized and identified as *S. haemolyticus* MST1, *P. aeruginosa* MST2, *K. pneumoniae* MST3, *E. coli* MST4, and *E. coli* MST5. Notably, both *E. coli* strains exhibited different behaviors in antibiotic susceptibility assays using the VITEK 2 system, in which *E. coli* MST4 was more virulent than *E. coli* MST5. *K. pneumoniae* MST3, *E. coli* MST4, and *E. coli* MST5 are extended-spectrum *β*-lactamases (ESBLs) producing bacteria. Moreover, *S. haemolyticus* MST1 emerged as a methicillin-resistant coagulase-negative *staphylococcus* (MRCoNS), whereas *P. aeruginosa* MST2 demonstrated great resistance to common bactericidal antibiotics. We are currently attempting to develop effective antibacterial wound dressings adopting these isolates as reliable and specific models.

## Author contributions

M.A.H. and T.M.T. conceived the project; M.A.H., S.A., and T.M.T. conducted the experiments; M.A.H. and T.M.T. analyzed the data and designed the figures; M.A.H., S.A., and T.M.T. participated in writing the draft of the manuscript; M.A.H. and T.M.T. revised and finalized the manuscript; M.A.H., S.A.E., H.M.E and T.M.T. supervised the study. All authors read the manuscript and approved the submitted version.

## Declaration of Competing Interest

The authors declare that they have no known competing financial interests or personal relationships that could have appeared to influence the work reported in this paper.
